# Risk of Major Cardiovascular Events in People with Down Syndrome

**DOI:** 10.1371/journal.pone.0137093

**Published:** 2015-09-30

**Authors:** Christopher G. Sobey, Courtney P. Judkins, Vijaya Sundararajan, Thanh G. Phan, Grant R. Drummond, Velandai K. Srikanth

**Affiliations:** 1 Cardiovacular Disease Program, Biomedicine Discovery Institute and Department of Pharmacology, Monash University, Clayton, Victoria, Australia; 2 Department of Surgery, Monash Medical Centre, Southern Clinical School, Monash University, Clayton, Victoria, Australia; 3 Stroke and Ageing Research Group, Department of Medicine, School of Clinical Sciences at Monash Health, Monash University, Clayton, Victoria, Australia; 4 Department of Medicine, St. Vincent's Hospital, University of Melbourne, Fitzroy, Victoria, Australia; 5 Stroke Unit, Monash Health, Melbourne, Australia; 6 Menzies Research Institute, Hobart, Tasmania, Australia; Osaka University Graduate School of Medicine, JAPAN

## Abstract

**Background:**

Improved medical care over more than five decades has markedly increased life expectancy, from 12 years to approximately 60 years, in people with Down syndrome (DS). With increased survival into late adulthood, there is now a greater need for the medical care of people with DS to prevent and treat aging-related disorders. In the wider population, acquired cardiovascular diseases such as stroke and coronary heart disease are common with increasing age, but the risks of these diseases in people with DS are unknown. There are no population-level data on the incidence of acquired major cerebrovascular and coronary diseases in DS, and no data examining how cardiovascular comorbidities or risk factors in DS might impact on cardiovascular event incidence. Such data would be also valuable to inform health care planning for people with DS. Our objective was therefore to conduct a population-level matched cohort study to quantify the risk of incident major cardiovascular events in DS.

**Methods and Findings:**

A population-level matched cohort study compared the risk of incident cardiovascular events between hospitalized patients with and without DS, adjusting for sex, and vascular risk factors. The sample was derived from hospitalization data within the Australian state of Victoria from 1993–2010. For each DS admission, 4 exact age-matched non-DS admissions were randomly selected from all hospitalizations within a week of the relevant DS admission to form the comparison cohort. There were 4,081 people with DS and 16,324 without DS, with a total of 212,539 person-years of observation. Compared to the group without DS, there was a higher prevalence in the DS group of congenital heart disease, cardiac arrhythmia, dementia, pulmonary hypertension, diabetes and sleep apnea, and a lower prevalence of ever-smoking. DS was associated with a greater risk of incident cerebrovascular events (Risk Ratio, RR 2.70, 95% CI 2.08, 3.53) especially among females (RR 3.31, 95% CI 2.21, 4.94) and patients aged ≤50 years old. The association of DS with ischemic strokes was substantially attenuated on adjustment for cardioembolic risk (RR 1.93, 95% CI 1.04, 3.20), but unaffected by adjustment for atherosclerotic risk. DS was associated with a 40–70% reduced risk of any coronary event in males (RR 0.58, 95% CI 0.40, 0.84) but not in females (RR 1.14, 95% CI 0.73, 1.77).

**Conclusions:**

DS is associated with a high risk of stroke, expressed across all ages. Ischemic stroke risk in DS appears mostly driven by cardioembolic risk. The greater risk of hemorrhagic stroke and lower risk of coronary events (in males) in DS remain unexplained.

## Introduction

### Background/rationale

Improved medical care over more than five decades has markedly increased life expectancy, from 12 years to approximately 60 years, in people with Down syndrome (DS) [1,2]. With increased survival into late adulthood, there is now a greater need for the medical care of people with DS individuals to prevent and treat aging-related disorders. In the wider population, acquired cardiovascular diseases such as stroke and coronary heart disease are common with increasing age, but the risks of these diseases in people with DS are unknown.

In DS, the occurrence of dementia is well known [3]. Congenital heart disease is also recognized as a manifestation of DS [4] and is associated with a greater risk of cardiogenic embolism [5]. There is limited evidence in DS regarding the prevalence of hypertension and atherosclerosis, key cardiovascular factors predisposing to major age-related cardiovascular events. In a small number of post-mortem analyses, each in no more than 15 patients with DS (mean ages of 51–56 at death), atherosclerotic lesion formation was shown to be much less in coronary arteries from patients with DS than those from non-DS controls [6–8]. Furthermore, blood pressure was reported to be lower in 70 people with DS (mean age 39 years) than in age- and sex-matched controls [6]. However, with increasing survival, the cardiovascular risk profile of people with DS may be changing. Adults with DS are more likely to be overweight or obese [9], and in one study, the prevalence of traditional risk factors has been reported to be similar to that in the general population [10,11]. However, there are no population-level data on the incidence of acquired major cerebrovascular and coronary diseases in people with DS, and no data examining how cardiovascular comorbidities or risk factors in DS might impact on cardiovascular event incidence. Such data would be also valuable to inform health care planning for people with DS.

### Objective

To conduct a population-level matched cohort study to quantify the risk of incident major cardiovascular events in DS.

## Methods

This study was approved by Monash University Human Research Ethics Committee (CF11/1920–2011001086). Consent was not obtained from patients. Patient records and information was anonymized and de-identified prior to analysis.

### Study Setting, Population, and Data Sources

The sample was derived from all public and private hospitalizations in Victoria, a State of Australia with a population >5 million (approximately 25% of the Australian population), included within the Victorian Admitted Episodes Dataset (VAED) in the 17-year period between 1 July 1993 and 30 June 2010. Australians have universal access to hospital medical care and all Victorian hospitals contribute to the VAED because the reporting of cases is mandatory and connected to hospital funding. The datasets contain demographic and clinical information on each episode of patient care. Diagnostic information is coded according to the International Statistical Classification of Diseases (ICD) and Related Health Problems. ICD-9-CM was used until June 1998 followed by the ICD-10 (10^th^ Revision), Australian Modification (ICD-10-AM), which is currently in use [12]. For each hospital admission, coding occurs at the conclusion of a patient’s episode of care when the entire medical record is reviewed by experienced clinical coders who record the diagnoses and procedures relevant to the admission. Admitted episode data are compiled by over 300 individual hospitals and maintained by the Victorian State Department of Health. The quality of Victoria’s hospital data is maintained using an independent audit program. The data does not contain identifying variables when released by the Victorian Department of Health for research use.

### Study Design and Size

We used a matched cohort design to compare patients with and without DS for the risk of incident cardiovascular events. Patients with DS were identified in the VAED for the study period 1 July 1993 and 30 June 2010, using ICD-9-CM/ICD-10 AM codes (7580/Q90). Based on the date of hospital admission, we then randomly selected 4 exact age-matched patients without DS from the VAED who were admitted to hospital within ±7 days of the index patient with DS. DS and non-DS cohorts were then followed for the outcome of first admission for any major cardiovascular event during the 17-year study period. The ascertainment of outcomes was passive using diagnostic coding from coded routinely collected hospital discharge data. Deaths occurring during the observation period were obtained from the Victorian Death Registry [13], but cause of death was not available.

### Cardiovascular Event Outcomes and Comorbidities

The main outcome events were the incidence of any cerebrovascular event (first admission for ischemic or hemorrhagic stroke, or transient ischemic attack [TIA]), or any coronary event (first admission for myocardial infarction [MI] or angina). Secondary individual outcomes were the incidence of ischemic stroke and TIA, hemorrhagic stroke, and MI. The ICD-9-CM/ICD-10 AM codes used for Ischemic Stroke, Hemorrhagic Stroke, TIA, MI, and Angina are listed in **Table A in S1 File**. The coding quality for cerebrovascular events and MI in Victorian public hospital data is very high for comparison between coding auditors and hospital coders (kappa = 0.91 for both types of event) [14]. The presence of the following concurrent cardiovascular risk factors and conditions was also identified for each person using relevant ICD-9-CM/ICD-10 AM codes (see **Table A in S1 File**); Hypertension, Diabetes, Smoking, Cardiac Arrhythmia (including Atrial Fibrillation), Sleep Apnea, Congenital Heart Disease, Pulmonary Hypertension, and Moyamoya Disease. To simplify expression of cardiovascular risk, a composite variable for cardioembolic stroke risk was generated if congenital heart disease, cardiac arrhythmia or pulmonary hypertension were present. Another composite variable was generated for atherosclerotic vascular risk combining the traditional vascular risk factors of hypertension, diabetes mellitus, smoking, and sleep apnea. Finally, an overall cardiovascular risk variable was generated combining the above variables and Moyamoya disease.

### Statistical Methods and Analyses

Stata (11.1, Stata Corporation, College Station, Texas, USA, 2006) was used for all statistical analyses. Given the matched cohort design, proportions of study factors were compared between DS and non-DS groups using McNemar test for paired samples. For the main analyses, Cox proportional hazards regression was considered but deemed inappropriate as it would have resulted in the loss of matching. Instead, we used the methods of generalized estimating equations, specifying a term for each DS/non-DS pair as the panel variable to preserve matching. Log binomial regressions were then performed to estimate the risk ratios for the associations between DS and incident cardiovascular events during the study period, adjusting for sex and the presence of concurrent cardiovascular risk. These analyses were repeated in males and females to explore potential sex differences. Additionally, post-hoc paired comparisons were conducted comparing proportions of cardiovascular events between DS and non-DS groups stratified by age group (≤ 18 years, 19–50 years, and >50 years) and sex.

## Results

### Sample characteristics

The sample is described in **Table 1** and comprised 4,081 patients with DS (52.8% male) and 16,324 patients without DS (49.1% male) individuals, with across all ages from 0–89 years (except ages 79, 82, and 85–87) and with mean age of first hospitalization in both groups of 18.5 years (SD 20.0). In total, there were 212,539 person-years of observation (46,850 person-years in DS and 165,689 person-years in non-DS groups). In each group there were 58.2% of individuals aged 0–18 years (2,375 with DS and 9,496 non-DS), 32.8% were aged 19–50 (1,337 with DS and 5,352 non-DS), and 9.0% were aged >50 years (369 with DS and 1,476 non-DS). A greater proportion of patients with DS (1,465, 35.9%) had any cardiovascular risk factor compared with those without DS (3,130, 19.2%) (P<0.001), mainly expressed as a difference in cardioembolic risk (congenital heart disease and cardiac arrhythmia) which was present in 1,048 (25.7%) of DS and 291 (1.8%) of non-DS groups. Overall, atherosclerotic risk was slightly less in DS, occurring in 591 (14.5%) individuals with DS compared with 3,002 (18.4%) non-DS individuals (P<0.001). However, among those aged ≤50 years, there was a significantly higher prevalence of diabetes mellitus, hypertension and sleep apnea (all P<0.001) in those with DS. Conversely, amongst those aged >50 years, there was a significantly lower prevalence of diabetes mellitus (P<0.05) and hypertension (P<0.001) in those with DS. The prevalence of smoking increased with age in both DS and non-DS groups, but was consistently higher in the non-DS group (P<0.001). Only patients with DS (7/4,081, 0.1%) were diagnosed with concurrent Moyamoya disease. As expected, the prevalence of dementia was significantly greater in the DS group, this difference being most prominent among those aged >50 years, but also present in those aged 19–50 years. The higher prevalence in the DS group of congenital heart disease, dementia, diabetes, pulmonary hypertension, hypothyroidism, and Moyamoya disease, and a lower prevalence of smoking were present in both males and females **(Tables B and C in S1 File),** whereas in females only, there was a significantly higher prevalence of cardiac arrhythmia in individuals with DS **(Table C in S1 File)**. More deaths occurred during the study period in the DS group (329, 8.1%) than in the non-DS group (195, 1.2%) (P<0.001).

**Table 1:** Sample characteristics. (Data are n [%] patients unless indicated otherwise).

Patient group (age range, years)	All DS (0–89)	All Non-DS (0–89)	DS (0–18)	Non-DS (0–18)	DS (19–50)	Non-DS (19–50)	DS (51+)	Non-DS (51+)
Whole sample	4,081	16,324	2,375	9,496	1,337	5,352	369	1,476
Males	2,153 (52.8)	8,019 (49.1)***	1,282 (54.0)	5,091 (53.6)	685 (51.2)	2,181 (40.7)***	186 (50.4)	747 (50.6)
**Comorbidities:**								
Any cardiac arrhythmia	80 (2.0)	202 (1.2)***	20 (0.8)	4 (0.04)***	24 (1.8)	50 (0.9)*	36 (9.8)	148 (10.0)
Congenital heart disease	975 (23.4)	90 (0.5)***	835 (35.2)	77 (0.8)***	120 (9.0)	9 (0.2)***	20 (5.4)	4 (0.3)***
Diabetes	145 (3.6)	322 (2.0)***	25 (1.1)	23 (0.2)***	87 (6.5)	93 (1.7)***	33 (8.9)	206 (14.0)**
Hypertension	107 (2.6)	549 (3.4) *	33 (1.4)	14 (0.1) ***	37 (2.8)	154 (2.9)	37 (10.0)	381 (25.8) ***
Pulmonary hypertension	153 (3.7)	8 (0.05) ***	111 (4.7)	4 (0.04) ***	37 (2.8)	2 (0.04) ***	5 (1.4)	2 (0.1) **
Sleep apnea	270 (6.6)	415 (2.5) ***	201 (8.5)	274 (2.9) ***	56 (4.2)	75 (1.4) ***	13 (3.5)	66 (4.5)
Smoking	142 (3.5)	2,300 (14.1) ***	2 (0.1)	149 (1.6) ***	90 (6.7)	1,537 (28.7) ***	50 (13.6)	614 (41.6) ***
Moyamoya disease	7 (0.1)	0 (0.0) ***	4 (0.2)	0 (0.0) **	3 (0.2)	0 (0.0) **	0 (0.0)	0 (0.0)
Any cardioembolic risk factor †	1,048 (25.7)	291 (1.8) ***	854 (36.0)	81 (0.8) ***	140 (10.5)	58 (1.1) ***	54 (14.6)	152 (10.3) *
Any atherosclerotic risk factor ††	591 (14.5)	3,002 (18.4) ***	253 (10.7)	455 (4.8) ***	237 (17.7)	1,694 (31.6) ***	101 (27.4)	853 (57.8) ***
Any cardiovascular risk factor †††	1,465 (35.9)	3,130 (19.2) ***	992 (41.8)	530 (5.6) ***	345 (25.7)	1,722 (32.2) ***	128 (34.7)	878 (59.5) ***
Hypothyroidism	198 (4.9)	38 (0.2) ***	75 (3.2)	8 (0.08) ***	75 (5.6)	17 (0.3) ***	48 (13.0)	13 (0.9) ***
Dementia	214 (5.2)	92 (0.6) ***	1 (0.04)	5 (0.05)	57 (4.3)	28 (0.5) ***	156 (42.3)	59 (4.0) ***

DS = Down syndrome.

† presence of either of congenital heart disease, cardiac arrhythmia or pulmonary hypertension

†† presence of any of hypertension, diabetes mellitus, sleep apnea, smoking

††† presence of any of congenital heart disease, cardiac arrhythmia, pulmonary hypertension, hypertension, diabetes mellitus, sleep apnea, smoking or Moyamoya disease

* P<0.05

** P<0.01

*** P<0.001 (McNemar test)–for comparing proportions between DS and non-DS groups

### Risk of Incident Cardiovascular Events in DS

A total of 88 patients with DS (2.2%) suffered 129 cerebrovascular events (stroke and/or TIA), whereas 129 patients without DS (0.8%) suffered 211 cerebrovascular events during the 17-year study period **(Tables 1–3; Table D in S1 File)**. Of the 88 patients with DS who suffered cerebrovascular events, 34 (38.6%) had multiple events (accounting for 98/148 events) compared with 49 (38.0%) in the non-DS individuals group who suffered multiple events (131/211 events). Mean age at first cerebrovascular event was 41.8 (SD 22.8) years in those with DS and 57.1 (SD 17.9) years in those without DS. Among those aged ≤50 years at first hospitalization, 46 (1.1%) individuals with DS, but only 34 (0.2%) people without DS, suffered a cerebrovascular event **(Table 2; Table D in S1 File)**. Amongst patients with DS, the following frequencies were observed for incident ischemic strokes (36/88, 41%), hemorrhagic strokes (25/88, 28%), strokes of unspecified type (40/88, 45%) and TIA (17/88, 19%). In the non-DS group, incident ischemic strokes (38/129, 29%) and incident hemorrhagic strokes (30/129, 23%) were less common, with the following frequencies observed for strokes of unspecified type (58/129, 45%) and TIA (34/129, 26%). Among those who died, a slightly lower proportion of patients with DS suffered a cerebrovascular event prior to death compared with those without DS individuals (6.4% versus 14.9%, p = 0.002).

**Table 2:** Major cardiovascular events in males. (Data are n [%] patients unless indicated otherwise).

Patient group (age range, years)	All DS (0–89)	All Non-DS (0–89)	DS (0–18)	Non-DS (0–18)	DS (19–50)	Non-DS (19–50)	DS (51+)	Non-DS (51+)
**Sample size**	2,153	8,019	1,282	5,091	685	2,181	186	747
**Outcome Events**								
**Any cerebrovascular event**	47 (2.2)	76 (1.0) ***	11 (0.9)	6 (0.1) ***	11 (1.6)	11 (0.5) **	25 (13.4)	59 (7.9) *
**Any stroke**	41 (1.9)	61 (0.7) ***	11 (0.9)	6 (0.1) ***	9 (1.3)	10 (0.5) *	21 (11.3)	45 (6.0) *
**Ischemic stroke**	17 (0.8)	24 (0.3) **	7 (0.5)	2 (0.04) ***	4 (0.6)	4 (0.2)	6 (3.2)	18 (2.4)
**Hemorrhagic stroke**	16 (0.7)	15 (0.2) ***	5 (0.4)	2 (0.04) **	4 (0.6)	6 (0.3)	7 (3.8)	7 (0.9)*
**Any coronary event**	31 (1.4)	201 (2.5) **	1 (0.1)	0 (0.0)	11 (1.6)	59 (2.7)	19 (10.2)	142 (19.0) **
**Myocardial infarction**	24 (1.1)	187 (2.3) ***	1 (0.1)	0 (0.0)	7 (1.0)	53 (2.4)	16 (8.6)	134 (17.9) **

DS = Down syndrome.

Any cerebrovascular event includes stroke (ischemic, hemorrhagic or unspecified) or transient ischemic attack.

Any coronary event includes myocardial infarction or angina.

* P<0.05

** P<0.01

*** P<0.001 (McNemar test)—for comparing proportions between DS and non-DS groups

**Table 3:** Major cardiovascular events in females. (Data are n [%] patients unless indicated otherwise).

Patient group (age range, years)	All DS (0–89)	All Non-DS (0–89)	DS (0–18)	Non-DS (0–18)	DS (19–50)	Non-DS (19–50)	DS (51+)	Non-DS (51+)
**Sample size**	1,928	8,305	1,093	4,405	652	3,171	183	729
**Outcome Events**								
**Any cerebrovascular event**	41 (2.1)	53 (0.6) ***	6 (0.5)	0 (0.0) ***	18 (2.8)	17 (0.5) ***	17 (9.3)	36 (4.9) *
**Any stroke**	36 (1.9)	44 (0.5) ***	6 (0.5)	0 (0.0) ***	15 (2.3)	16 (0.5) ***	15 (8.2)	28 (3.8) *
**Ischemic stroke**	19 (1.0)	14 (0.2) ***	5 (0.5)	0 (0.0)***	11 (1.7)	6 (0.2) ***	3 (1.6)	8 (1.1)
**Hemorrhagic stroke**	9 (0.5)	15 (0.2) *	0 (0.0)	0 (0.0)	3 (0.5)	7 (0.2)	6 (3.3)	8 (1.1) *
**Any coronary event**	24 (1.2)	89 (1.1)	2 (0.2)	1 (0.02)	6 (0.9)	15 (0.5)	16 (8.7)	73 (10.0)
**Myocardial infarction**	20 (1.0)	72 (0.9)	2 (0.2)	1 (0.02)	4 (0.6)	8 (0.25)	14 (7.7)	63 (8.6)

DS = Down syndrome

Any cerebrovascular event includes stroke (ischemic, hemorrhagic or unspecified) or transient ischemic attack.

Any coronary event includes myocardial infarction or angina.

* P<0.05

** P<0.01

*** P<0.001 (McNemar test)—for comparing proportions between DS and non-DS groups

A total of 55 patients with DS (1.3%) suffered 118 coronary events (MI and/or angina), whereas 290 individuals without DS (1.8%) suffered 659 coronary events during the 17-year study period **(Tables 2–4; Table D in S1 File)**. Of the 55 patients with DS who suffered coronary events, 20 (36.4%) had multiple events (accounting for 83/118 events) compared with 145 (50.0%) in the non-DS group (514/659 events). Mean age at first coronary event was 53.7 (SD 19.2) years in those with DS and 58.3 (SD 12.1) years in those without DS. Amongst patients with DS, the following frequencies were observed for incident MI (44/55, 80%) and angina (23/55, 42%), compared with the non-DS group, incident MI (259/290, 89%) and angina (120/290, 41%). Among those who died, far fewer patients with DS suffered a coronary event than those without DS (5.8% versus 16.9%, p<0.001).

**Table 4:** Risk of cardiovascular events in patients with Down Syndrome. (Data are n [%] patients unless otherwise indicated).

	DS (n = 4,081)	Non-DS (n = 16,324)	Model 1 Risk Ratio (95% CI)	Model 2 Risk Ratio (95% CI)
**Cerebrovascular event**	**88 (2.2)**	**129 (0.8)**	**2.70 (2.08–3.53)** ***	**1.95 (1.49–2.56)** ***
Stroke	77 (1.9)	105 (0.6)	2.91 (2.18–3.88)***	2.09 (1.55–2.80)***
Ischemic stroke	36 (0.9)	38 (0.2)	3.76 (2.39–5.92)***	2.59 (1.63–4.11)***
Hemorrhagic stroke	25 (0.6)	30 (0.2)	3.31 (1.95–5.60)***	2.67 (1.56–4.57)***
**Coronary event**	**55 (1.3)**	**290 (1.8)**	**0.74 (0.57–0.98)** *	**0.47 (0.35–0.62)** ***
MI	44 (1.1)	259 (1.6)	0.67 (0.49–0.91)**	0.42 (0.30–0.57)***

DS = Down Syndrome; MI = myocardial infarction; TIA = transient ischemic attack.

Cerebrovascular events include stroke (ischemic, hemorrhagic or unspecified) or transient ischemic attack.

Coronary events include myocardial infarction or angina.

Model 1: adjusted for sex

Model 2: adjusted for sex and overall cardiovascular risk

(Overall cardiovascular risk is represented by a variable that includes the presence of any of congenital heart disease, cardiac arrhythmia, pulmonary hypertension, hypertension, diabetes mellitus, sleep apnea, smoking or Moyamoya disease)

* P<0.05

** P<0.01

*** P<0.001

In multivariable regression adjusting for sex **(Model 1, Table 4**), DS was associated with a greater risk of any incident cerebrovascular event (Risk Ratio, RR 2.70, 95% CI 2.08, 3.53), stroke (RR 2.91, 95% CI 2.18, 3.88), ischemic stroke (RR 3.76, 95% CI 2.39, 5.92), and hemorrhagic stroke (RR 3.31, 95% CI 1.95, 5.60). These associations were attenuated but remained significant after adjusting for the overall cardiovascular risk term **(Model 2, Table 4)**. The association of DS with ischemic stroke was substantially attenuated (RR 1.93, 95% CI 1.04, 3.20) when adjusted for cardioembolic risk, but was strengthened by adjustment for atherosclerotic risk (RR 4.19, 95% CI 2.66, 6.60). The association of DS with hemorrhagic stroke was largely unchanged by the adjustment for either cardioembolic (RR 3.04, 95% CI 1.71, 5.42) or atherosclerotic risk (RR 3.59, 95% CI 2.12, 6.09). Adjusting for sex, DS was associated with a lower risk of any coronary event or myocardial infarction, and these were strengthened after adjusting for overall cardiovascular risk **(Models 1 and 2, Table 4).**

In males, **(Model 1, Table E in S1 File**), DS was associated with a greater risk of any cerebrovascular event (RR 2.32, 95% CI 1.63, 3.30), stroke (RR 2.51, 95% CI 1.70, 3.69), ischemic stroke (RR 2.63, 95% CI 1.42, 4.87) or hemorrhagic stroke (RR 4.02, 95% CI 2.00, 8.10). In females **(Model 1, Table E in S1 File**), the magnitude of the associations were larger than in males for cerebrovascular events (RR 3.31, 95% CI 2.21, 4.94), stroke (RR 3.52, 95% CI 2.27, 5.45), and ischemic stroke (RR 5.84, 95% CI 2.93, 11.64). Adjustment for cardioembolic risk completely attenuated the association of DS with ischemic stroke in males (RR 0.79, 95% CI 0.37, 1.66), and less so in females (RR 4.83, 95% CI 2.24, 10.38). Adjustment for atherosclerotic risk marginally strengthened the association of DS with ischemic stroke in either sex (data not shown). Compared with their counterparts in the non-DS group, cerebrovascular events occurred in similarly greater frequency among male and female patients with DS, albeit with very low numbers in the strata. The risk of any coronary event or MI was significantly lower amongst male DS but not female patients with DS, even after adjusting for overall cardiovascular risk **(Table E in S1 File)** or atherosclerotic risk (data not shown).

## Discussion

### Key results

This large-scale study provides estimates of the risk of major cardiovascular events in patients with DS. We found that patients with DS were at high risk of cerebrovascular events, but at a lower risk of coronary events (males only), compared with age-matched individuals with no DS. The increased risk for cerebrovascular events was seen for both ischemic and hemorrhagic strokes in all age groups, and was generally higher in females than males. The association between DS and ischemic stroke appeared to be explained largely by cardioembolic risk.

DS is associated with a high risk of congenital heart disease, particularly atrioventricular septal defects [15]. There is a high risk of stroke associated with congenital heart disease, mainly due to cardioembolism [5]. Procedures performed for congenital heart disease such as cardiopulmonary bypass, cardiac surgery (e.g. mechanical valve) and catheterisation serve as markers for increased future risk of stroke, possibly reflecting a heightened risk of thromboembolism [5]. Individuals with DS in our study had significantly greater diagnoses of congenital heart disease, associated pulmonary hypertension, and cardiac arrhythmia, conditions representing high cardioembolic risk. Indeed, adjustment for the cardioembolic risk term markedly attenuated the association between DS and ischemic stroke, suggesting a mediating influence. Our findings that the risk of stroke in DS was present even in those aged ≤50 years also supports this hypothesis. The greater risk of hemorrhagic strokes in DS may have alternative explanations. Firstly, cardioembolic infarcts may often undergo hemorrhagic transformation, and this may be misclassified as hemorrhagic stroke. Alternatively, patients with congenital heart disease and arrhythmia may be anticoagulated which is an additional risk factor for hemorrhage. We were unable to explore these aspects in detail, and they deserve investigation in future research. We found a higher prevalence of traditional atherosclerotic risk factors (barring smoking) in patients with DS ≤18 years of age. These differences were small in absolute terms, and some (e.g. hypertension) may be closely linked with cardiovascular abnormalities such as common artrioventricular canal, atrial septal defect, ventricular septal defect, patent foramen ovale, and patent ductus arteriosus, and hence quite different in pathogenesis from hypertension seen in the general population. These differences were reversed among those >18 years reflecting either the greater accrual of such risk after mid-life in the general population, poorer survival of patients with DS beyond middle age, or both. However, the associations of DS with ischemic stroke appeared independent of traditional atherosclerotic risk assigned by these factors. A reasonable explanation for this may be that diagnostic coding in hospital administrative datasets is insufficient to fully characterise cardiovascular risk, and hence the true adjusted estimate of stroke risk may be different from our estimates. An uncommon, but previously reported cause of stroke in DS is the occurrence of Moyamoya disease, a chronic vascular occlusive condition involving the internal carotid artery [16,17]. We found 7 patients with both DS and Moyamoya disease, all of whom had strokes, whereas none of those in the non-DS group had the condition. Although rare, Moyamoya disease thus could explain some of the stroke risk not quite explained by other cardiovascular factors. A relatively surprising finding was that DS was associated with a lower risk of coronary events in males, independent of vascular risk factor profile. This is consistent with previous post-mortem findings that patients with DS have markedly reduced atherosclerotic lesion formation in coronary vessels compared with patients without DS [6–8], but the biological reason for this is unknown. We were unable to investigate whether patients with DS had undergone cardiovascular surgery, and hence were unable to stratify risk of cardiovascular events based on whether or not they had undergone surgical repair.

This study has several strengths. It is the first study examining the risk of major cardiovascular events in people with DS at a population level. Our sample size was large with a substantial period of observation, allowing for the fact that DS is a relatively rare disorder with an estimated 1:1,100 (0.09%) of Australian babies born with Down Syndrome [18] and 12.6 per 10,000 live births (~0.13%) in the United States of America [19]. Given that the risk of stroke and MI are heavily influenced by age, we matched 1:4 exactly on age to remove its confounding influence [20]. We additionally controlled for the influence of other vascular factors and attempted to partition the influence of cardioembolic and atherosclerotic factors on cerebrovascular and coronary event risk. We used well-audited and standard methods of diagnostic coding to identify DS and outcomes, with established data linkage procedures.

### Limitations

There are also potential limitations to this study. The use of diagnostic coding to define exposure or outcome may be subject to a risk of misclassification. However, the risk of such error is likely to be very low in the diagnosis of a rare chromosomal disorder such as DS, and indeed our data demonstrates excellent consistency and direction of associations of DS with known factors such as age, congenital heart disease, and dementia. The risk of misclassification may be somewhat greater for cardiovascular events. Diagnostic coding for stroke and MI has a high positive predictive value but less sensitivity when compared with gold-standard clinical neurologist diagnosis [13,21–23]. Change in coding systems from ICD-9 to ICD-10 may cause variation in exposure or outcome detection over time periods. However, there were strong associations in the expected directions between vascular risk terms and cardiovascular outcome events in our overall sample (data not shown) providing some support for appropriate risk factor and outcome classification in general. In a previous report using hospital administrative data, it was found that admissions for DS with congenital heart disease decreased over time in contrast to non-DS admissions for congenital heart disease, raising the possibility of systematic bias in recording health information in relation to DS admissions [24]. If this were the case, then there may be unmeasured confounding in our observed associations between DS and vascular risk factors. However, if such misclassification were similar (non-differential) in those with and without DS, this may have led to an underestimation of associations. Moreover, we may not have ascertained all patients with DS admitted to hospital over our study period if their DS status was missed during coding. Under-ascertainment of DS, if present, may reduce the generalizability of our results. A proportion of strokes recorded in our study were of unspecified type, and therefore our estimates of risk for stroke types may be subject to some error. Moreover, because of the nature of the data, we do not have confirmation using brain scans regarding the exact stroke type, or possible cardiac interventional procedures that may have occurred, limiting our inferences about stroke mechanisms. Our study only deals with patients who were hospitalized or received Emergency Department care in hospitals. However, the large statewide coverage of hospitals in our linkage dataset ensures generalizability to the majority of the population. Finally, given the small number of outcome events relative to the sample size, we could not assess the individual contribution of cardiovascular risk factors to the risk of outcomes or perform multivariable regression analyses in age-stratified subgroups. However, we adjusted for a composite measure of cardiovascular risk in our multivariable analysis, and supplemented this by performing post-hoc exploration of patient characteristics and cardiovascular events in age- and sex-stratified subgroups.

### Interpretation

Our study brings to attention for the first time the high risk of incident cerebrovascular events in people with DS. The risk of ischemic stroke in DS is expressed across all ages, and potentially explained by the presence of conditions such as congenital heart disease, cardiac arrhythmia and Moyamoya disease. The reasons for the increased risk of hemorrhagic stroke in DS, and lower risk of coronary events in males with DS deserve further study.

## Supporting Information

S1 File International Classification of Disease Codes used for Analyses (Table A). Males: sample characteristics (Table B). Females: sample characteristics **(Table C).** Major cardiovascular events (Table D). Risk of major cardiovascular events in patients with Down Syndrome stratified by sex **(Table E).**
(DOCX)
