# Supplementary material for: Risk of Major Cardiovascular Events in People with Down Syndrome
Source: PLoS One. 2015 Sep 30;10(9):e0137093. doi: 10.1371/journal.pone.0137093 (PMC4589343; doi:10.1371/journal.pone.0137093)
Supplement: S1 File — Males: sample characteristics (Table B). Females: sample characteristics (Table C). Major cardiovascular events (Table D). Risk of major cardiovascular events in patients with Down Syndrome stratified by sex (Table E). (DOCX) [file pone.0137093.s001.docx]

**S1 SUPPORTING INFORMATION**

**Risk of Major Cardiovascular Events in People with Down Syndrome**^1,2^Christopher G. Sobey, PhD, FAHA*

^1^Courtney P. Judkins, PhD*

^3,4^Vijaya Sundararajan, MD, MPH

^3,5^Thanh G. Phan, MBBS, PhD

^1,2^Grant R. Drummond, PhD

^3,5,6^Velandai K. Srikanth, MBBS, PhD, FRACP

^1^ Cardiovacular Disease Program, Biomedicine Discovery Institute and Department of Pharmacology, Monash University, Clayton, Victoria, Australia.

^2^ Department of Surgery, Monash Medical Centre, Southern Clinical School, Monash University, Clayton, Victoria, Australia.

^3^ Stroke and Ageing Research Group, Department of Medicine, School of Clinical Sciences at Monash Health, Monash University, Clayton, Victoria, Australia.

^4^ Department of Medicine, St. Vincent's Hospital, University of Melbourne, Fitzroy, Victoria, Australia.

^5^ Stroke Unit, Monash Health, Melbourne, Australia.

^6^ Menzies Research Institute, Hobart, Tasmania, Australia.

*Equal contributions.

**Short title:** Cardiovascular events in Down Syndrome

**Send correspondence to:**Prof. Christopher G. Sobey, Ph.D., Department of Pharmacology, Monash University,
Wellington Road, Clayton, Victoria 3800, AUSTRALIA.
Tel: +61 3 9905-4189; Fax: +61 3 9902-9500; e-mail: [chris.sobey@monash.edu](mailto:chris.sobey@monash.edu)

**Supplemental Tables**

**S1 Table A. International Classification of Disease Codes used for Analyses**

| **Event/Comorbidity** | **ICD-9-CM Codes**  **July 1993-June 1998** | **ICD-10-AM Codes**  **July 1998-June 2010** |
| --- | --- | --- |
| Angina | 4111 | I200 |
| Atrial Fibrillation | 42731 | I48 |
| Cardiac Arrhythmia | 42741, 42761, 42769, 42781, 42789, 4279 | I49 |
| Cigarette Smoking | V6542, V1583, V170, V1582, 98984 | Z716, Z720, Z812, Z8643, T652 |
| Congenital Heart Disease | 745, 746 | Q20, Q21, Q22, Q23, Q24 |
| Dementia | 2900, 2901, 2904, 2940, 2941, 2948, 2949, 2930, 2938, 310 | F00, F01, F02, F03, F04, F05, F06, F07, F09 |
| Diabetes | 250 | E10, E11, E13, E14 |
| Down Syndrome | 7580 | Q90 |
| Hemorrhagic Stroke | 430, 431, 432 | I60, I61, I62 |
| Hypertension | 4019, 4029, 4039, 4049, 4059 | I10, I11, I12, I13, I15 |
| Hypothyroidism | 244 | E03 |
| Ischemic Stroke | 43391, 43401, 43411, 43491, 4376 | I63 |
| Moyamoya Disease | 4375 | I675 |
| Myocardial Infarction (MI) | 4101, 4104, 4107, 4108, 4109, 4230, 4295, 4297 | I21, I22, I23, I24, I25 |
| Pulmonary hypertension | 4160 | I270 |
| Sleep Apnea | 78050, 78051, 78059, 347 | G473, G474, G478, G479 |
| Transient Ischemic Attack (TIA) | 435 | G450, G451, G452, G453, G458, G459 |
| Unspecified Stroke | 433,  434,  435,  436,  4370,   4371,  4372,  4373, 4374, 4376, 4379 | G46, I64, I65, I66, I670, I671, I672, I673, I674, I676, I678, I679 |

**S1 Table B – Males: sample characteristics (Data are n [%] patients unless otherwise indicated)**

| **Patient group  (age range, years**) | **All DS (0-89)** | **All Non-DS (0-89)** | **DS (0-18)** | **Non-DS (0-18)** | **DS (19-50)** | **Non-DS (19-50)** | **DS (51+)** | **Non-DS (51+)** |
| --- | --- | --- | --- | --- | --- | --- | --- | --- |
| Sample size | 2,153 (52.8) | 8,019 (49.1)*** | 1,282  (54.0) | 5,091 (53.6) | 685  (51.2) | 2,181 (40.7)*** | 186 (50.4) | 747 (50.6) |
| Mean age  (years; SD) | 18.0 (19.9) | 17.1 (20.3) | 3.4  (5.2) | 3.2 (5.0) | 34.3  (8.6) | 35.4 (8.6) | 58.2 (6.2) | 58.3 (6.7) |
|  |  |  |  |  |  |  |  |  |
| **Comorbidities:** |  |  |  |  |  |  |  |  |
| Any Cardiac Arrhythmia | 38 (1.8) | 129 (1.6) | 7 (1.3) | 3 (0.06)*** | 9 (1.3) | 35 (1.6) | 22 (11.8) | 91 (12.2) |
| Congenital Heart Disease | 491 (22.8) | 55 (0.7)*** | 418 (32.6) | 48 (0.9)*** | 62 (9.1) | 4 (0.2)*** | 11 (5.9) | 3 (0.4)*** |
| Diabetes | 66 (3.1) | 180 (2.2)* | 11 (0.9) | 14 (0.3)** | 40 (5.8) | 51 (2.3)*** | 15 (8.1) | 115 (15.4)** |
| Hypertension | 55 (2.6) | 289 (3.6)* | 14 (1.1) | 9 (0.2)*** | 21 (3.1) | 81 (3.7) | 20 (10.8) | 199 (26.6)*** |
| Pulmonary hypertension | 70 (3.2) | 5 (0.06)*** | 53 (4.1) | 3 (0.06)*** | 14 (2.0) | 1 (0.04)*** | 3 (1.6) | 1 (0.1)* |
| Sleep Apnea | 157 (7.3) | 258 (3.2)*** | 117 (9.1) | 159 (3.1)*** | 32 (4.7) | 52 (2.4)** | 8 (4.3) | 47 (6.3) |
| Smoking | 80 (3.7) | 1,145 (14.3)*** | 1 (0.1) | 60 (1.2)*** | 49 (7.1) | 706 (32.4)*** | 30 (16.1) | 379 (50.7)*** |
| Moyamoya Disease | 2 (0.1) | 0 (0.0)* | 2 (0.2) | 0 (0.0)* | 0 (0.0) | 0 (0.0) | 0 (0.0) | 0 (0.0) |
| Any cardioembolic risk factor † | 528 (24.5) | 183 (2.3)*** | 428 (33.4) | 52 (1.0)*** | 70 (10.2) | 38 (1.7)*** | 30 (16.1) | 93 (12.4) |
| Any atherosclerotic risk factor †† | 321 (14.9) | 1,514 (18.9)*** | 139 (10.8) | 238 (4.7)*** | 126 (18.4) | 791 (36.3)*** | 56 (30.1) | 485 (64.9)*** |
| Any cardiovascular risk factor ††† | 754 (35.0) | 1,593 (19.9)*** | 506 (39.4) | 287 (5.6)*** | 178 (26.0) | 807 (37.0)*** | 70 (37.6) | 499 (66.8)*** |
| Hypothyroidism | 88 (4.2) | 8 (0.1)*** | 46 (3.6) | 4 (0.08)*** | 28 (4.1) | 1 (0.05)*** | 14 (7.5) | 3 (0.4)*** |
| Dementia | 100 (4.6) | 49 (0.6)*** | 0 (0.0) | 3 (0.06) | 29 (4.2) | 19 (0.9)*** | 71 (38.2) | 27 (3.6)*** |

DS= Down syndrome.

† presence of either of congenital heart disease, cardiac arrhythmia or pulmonary hypertension

†† presence of any of hypertension, diabetes mellitus, sleep apnea, smoking

††† presence of any of congenital heart disease, cardiac arrhythmia, pulmonary hypertension, hypertension, diabetes mellitus, sleep apnea, smoking or Moyamoya disease

* P<0.05; ** P<0.01; *** P<0.001 (McNemar test) – for comparing proportions between DS and non-DS groups

**S1 Table C – Females: sample characteristics (Data are n [%] patients unless otherwise indicated)**

| **Patient group  (age range, years**) | **All DS (0-89)** | **All Non-DS (0-89)** | **DS (0-18)** | **Non-DS (0-18)** | **DS (19-50)** | **Non-DS (19-50)** | **DS (51+)** | **Non-DS (51+)** |
| --- | --- | --- | --- | --- | --- | --- | --- | --- |
| Sample size | 1,928 (47.2) | 8,305 (50.9)*** | 1,093  (46.0) | 4,405 (46.4) | 652  (48.8) | 3,171 (59.3)*** | 183 (49.6) | 729 (49.4) |
| Mean age  (years; SD) | 19.1 (20.2) | 19.9 (19.7) | 3.5  (5.3) | 3.7 (5.6) | 34.3  (8.3) | 33.6 (8.3) | 58.4 (7.7) | 58.3 (7.3) |
|  |  |  |  |  |  |  |  |  |
| **Comorbidities:** |  |  |  |  |  |  |  |  |
| Any Cardiac Arrhythmia | 42 (2.2) | 73 (0.9)*** | 13 (1.2) | 1 (0.02)*** | 15 (0.8) | 15 (0.5)*** | 14 (7.7) | 57 (7.8) |
| Congenital Heart Disease | 484 (25.1) | 35 (0.4)*** | 417 (38.2) | 29 (0.7)*** | 58 (8.9) | 5 (0.2)*** | 9 (4.9) | 1 (0.1)*** |
| Diabetes | 79 (4.1) | 142 (1.7)*** | 14 (1.3) | 9 (0.2)*** | 47 (7.2) | 42 (1.3)*** | 18 (9.8) | 91 (12.5) |
| Hypertension | 52 (2.7) | 260 (3.1) | 19 (1.7) | 5 (0.1)*** | 16 (2.5) | 73 (2.3) | 17 (9.3) | 182 (25.0)*** |
| Pulmonary hypertension | 83 (4.3) | 3 (0.04)*** | 58 (5.3) | 1 (0.02)*** | 23 (3.5) | 1 (0.03)*** | 2 (1.1) | 1 (0.1) |
| Sleep Apnea | 113 (5.9) | 157 (1.9)*** | 84 (7.7) | 115 (2.6)*** | 24 (3.7) | 23 (0.7)*** | 5 (2.7) | 19 (2.6) |
| Smoking | 62 (3.2) | 1,155 (13.9)*** | 1 (0.1) | 89 (2.0)*** | 41 (6.3) | 831 (26.2)*** | 20 (10.9) | 235 (32.2)*** |
| Moyamoya Disease | 5 (0.3) | 0 (0.0)*** | 2 (0.2) | 0 (0.0)* | 3 (0.5) | 0 (0.0)** | 0 (0.0) | 0 (0.0) |
| Any cardioembolic risk factor † | 520 (27.0) | 108 (1.3)*** | 426 (39.0) | 29 (0.7)*** | 70 (10.7) | 20 (0.6)*** | 24 (13.1) | 59 (8.1)* |
| Any atherosclerotic risk factor †† | 270 (14.0) | 1,488 (17.9)*** | 114 (10.4) | 217 (4.9)*** | 111 (17.0) | 903 (28.5)*** | 45 (24.6) | 368 (50.5)*** |
| Any cardiovascular risk factor ††† | 711 (36.9) | 1,537 (18.5)*** | 486 (44.5) | 243 (5.5)*** | 167 (25.6) | 915 (28.9) | 58 (31.7) | 379 (52.0)*** |
| Hypothyroidism | 110 (5.7) | 30 (0.4)*** | 29 (2.7) | 4 (0.1)*** | 47 (7.2) | 16 (0.5)*** | 34 (18.6) | 10 (1.4)*** |
| Dementia | 114 (5.9) | 43 (0.5)*** | 1 (0.1) | 2 (0.04) | 28 (4.3) | 9 (0.3)*** | 85 (46.4) | 32 (4.4)*** |

DS= Down syndrome.

† presence of either of congenital heart disease, cardiac arrhythmia or pulmonary hypertension

†† presence of any of hypertension, diabetes mellitus, sleep apnea, smoking

††† presence of any of congenital heart disease, cardiac arrhythmia, pulmonary hypertension, hypertension, diabetes mellitus, sleep apnea, smoking or Moyamoya disease

* P<0.05; ** P<0.01; *** P<0.001 (McNemar test) – for comparing proportions between DS and non-DS groups

**S1 Table D – Major cardiovascular events.** (Data are n [%] patients unless indicated otherwise)

| **Event** | **All DS (0-89)** | **All Non-DS (0-89)** | **DS (0-18)** | **Non-DS (0-18)** | **DS (19-50)** | **Non-DS (19-50)** | **DS (51+)** | **Non-DS (51+)** |
| --- | --- | --- | --- | --- | --- | --- | --- | --- |
| **Sample size** | 4,081 | 16,324 | 2,375 | 9,496 | 1,337 | 5,352 | 369 | 1,476 |
|  |  |  |  |  |  |  |  |  |
| **Any Cerebrovascular Event** | 88 (2.2) | 129 (0.8)*** | 17 (0.7) | 6 (0.06)*** | 29 (2.2) | 28 (0.5)*** | 42 (11.4) | 95 (6.4)** |
| **Any Stroke** | 77 (1.9) | 105 (0.6)*** | 17 (0.7) | 6 (0.06)*** | 24 (1.8) | 26 (0.5)*** | 36 (9.8) | 73 (5.0)*** |
| **Ischemic Stroke** | 36 (0.9) | 38 (0.2)*** | 12 (0.5) | 2 (0.02)*** | 15 (1.1) | 10 (0.2)*** | 9 (2.4) | 26 (1.8) |
| **Hemorrhagic Stroke** | 25  (0.6) | 30 (0.2)*** | 5 (0.2) | 2 (0.02)** | 7 (0.5) | 13 (0.2) | 13 (3.5) | 15 (1.0)*** |
|  |  |  |  |  |  |  |  |  |
| **Any Coronary Event** | 55  (1.3) | 290 (1.8) | 3 (0.1) | 1 (0.01)* | 17 (1.3) | 74 (1.4) | 35 (9.5) | 215 (14.6*) |
| **Myocardial Infarction** | 44  (1.1) | 259 (1.6)* | 3 (0.1) | 1 (0.01)* | 11 (0.8) | 61 (1.1) | 30 (8.1) | 197 (13.3)** |

DS= Down syndrome

* P<0.05; ** P<0.01; *** P<0.001 (McNemar test) – for comparing proportions between DS and non-DS groups

**S1 Table E. Risk of major cardiovascular events in patients with Down Syndrome stratified by sex (Data are n,% patients unless otherwise indicated)**

|  | **Males** | | | | **Females** | | | |
| --- | --- | --- | --- | --- | --- | --- | --- | --- |
|  | **DS**  (n=2,153) | **Non-DS**  (n=8,019) | **Model 1**  Risk Ratio  (95% CI) | **Model 2**  Risk Ratio  (95% CI) | **DS**  (n=1,928) | **Non-DS**  (n=8,305) | **Model 1**  Risk Ratio  (95% CI) | **Model 2**  Risk Ratio  (95% CI) |
| **Cerebrovascular events** | **47  (2.2)** | **76**  **(1.0)** | **2.32 (1.63-3.30)***** | **1.69 (1.18-2.43)**** | **41 (2.2)** | **53 (0.6)** | **3.31**  **(2.21-4.94)***** | **2.30 (1.52-3.47)***** |
| Stroke | 41  (1.9) | 61 (0.8) | 2.51 (1.70-3.69)*** | 1.83 (1.23-2.72)** | 36 (1.9) | 44 (0.5) | 3.52  (2.27-5.45)*** | 2.38 (1.52-3.71)*** |
| Ischemic stroke | 17  (0.8) | 24 (0.3) | 2.63 (1.42-4.87)** | 1.72 (0.92-3.21) | 19  (0.10) | 14 (0.2) | 5.84 (2.93-11.64)*** | 4.29 (2.12-8.70)*** |
| Hemorrhagic stroke | 16  (0.7) | 15 (0.2) | 4.02 (2.00-8.10)*** | 3.34 (1.64-6.82)*** | 9  (0.5) | 15 (0.2) | 2.58 (1.13-5.89)* | 1.93 (0.83-4.50) |
|  |  |  |  |  |  |  |  |  |
| **Coronary events** | **31  (1.4)** | **201 (2.5)** | **0.58 (0.40-0.84)**** | **0.36 (0.25-0.53)***** | **24  (1.2)** | **89 (1.1)** | **1.14 (0.73-1.77)** | **0.67 (0.43-1.04)** |
| Myocardial infarction | 24  (1.1) | 187 (2.3) | 0.49 (0.32-0.73)*** | 0.30 (0.20-0.46)*** | 20  (1.1) | 72 (0.9) | 1.15 (0.71-1.87) | 0.68 (0.42-1.11) |
|  |  |  |  |  |  |  |  |  |

DS= Down Syndrome; MI= myocardial infarction; TIA= transient ischemic attack.

Cerebrovascular events include stroke (ischemic, hemorrhagic or unspecified) or transient ischemic attack.

Coronary events include myocardial infarction or angina.

Model 1: unadjusted

Model 2: adjusted for overall cardiovascular risk

(Overall cardiovascular risk is represented by a variable that includes the presence of any of congenital heart disease, cardiac arrhythmia, pulmonary hypertension, hypertension, diabetes mellitus, sleep apnea, smoking or Moyamoya disease)

* P<0.05, ** P<0.01, *** P<0.001
